# Cognitive and mental health significantly contribute to disability in people ageing with HIV in Asia: an observational case‐control study

**DOI:** 10.1002/jia2.70052

**Published:** 2025-10-28

**Authors:** Grace Lui, Yaokai Chen, Chien‐Ching Hung, Pui Li Wong, Chen Seong Wong, Jason Leung, Xiaolei Xu, Catherine Cheung, Guanlin Li, Vivian Wong, Shui Shan Lee, Timothy Kwok, Reena Rajasuriar

**Affiliations:** ^1^ Department of Medicine and Therapeutics The Chinese University of Hong Kong Sha Tin Hong Kong SAR; ^2^ SH Ho Research Centre for Infectious Diseases The Chinese University of Hong Kong Sha Tin Hong Kong SAR; ^3^ Chongqing Public Health Medical Centre Chongqing China; ^4^ Department of Internal Medicine National Taiwan University Hospital Yunlin Branch Yunlin Taiwan; ^5^ Department of Internal Medicine National Taiwan University Hospital and National University Hospital College of Medicine Taipei Taiwan; ^6^ Department of Tropical Medicine and Parasitology National University Hospital College of Medicine Taipei Taiwan; ^7^ Centre of Excellence for Research in AIDS Department of Medicine Universiti Malaya Kuala Lumpur Malaysia; ^8^ Department of Medicine Faculty of Medicine Universiti Malaya Kuala Lumpur Malaysia; ^9^ National Centre for Infectious Diseases Singapore Singapore; ^10^ Jockey Club Centre for Osteoporosis Care and Control The Chinese University of Hong Kong Sha Tin Hong Kong SAR

**Keywords:** Asia, cognition, comorbidity, depression, disability, social isolation

## Abstract

**Introduction:**

Disability disproportionally impacts people living with HIV (PLWH). The burden and determinants of disability among PLWH in Asia have not been well studied.

**Methods:**

We conducted a multi‐country observational cross‐sectional study in five cities in Asia involving PLWH and age‐ and sex‐matched controls living without HIV from March 2020 to November 2023. We compared the prevalence of disability (measured by World Health Organization Disability Assessment Schedule 2.0, WHODAS 2.0) between PLWH and controls, and determined the association between living with HIV and disability using multivariable logistic regression and mediation analysis.

**Results:**

A total of 1004 PLWH and 416 age‐ and sex‐matched controls were enrolled. PLWH (mean age 53.6 ± 10.3 years, 84.4% male, 72.2% ≥1 comorbidities) had a higher Charlson Comorbidity Index, more depression, anxiety, stress, social isolation and loneliness, and poorer cognitive performance.

The prevalence of disability was 50.9% among PLWH and 40.6% among controls (*p*<0.001). PLWH had significantly higher WHODAS 2.0 complex score, and significantly more PLWH had impairments in all of the six domains of disability. The presence of disability correlated with living with HIV after adjusting for demographic characteristics, physical health parameters and cognition, but not after adjusting for socio‐behavioural variables and mental health parameters. Mediation analysis showed that living with HIV had a significant indirect effect on disability mediated by social isolation, mental health disorders and poor cognitive performance.

**Conclusions:**

PLWH in Asia had a higher burden of disability as compared with matched controls. The effect of living with HIV on disability was mediated by social isolation, mental health disorders and impaired cognition. Future work should be directed to developing interventions that mitigate these conditions with the goal of reducing disability among PLWH.

## 1 INTRODUCTION

Disability is an important health outcome among people living with HIV (PLWH), but is often neglected in HIV programmes [1]. Disability signifies difficulty or dependence in performing activities needed for independent living, while frailty is a physiologic state of increased vulnerability to stressors due to decreased physiologic reserves [2]. The process from robustness to disability is a continuum with frailty regarded as a preceding stage leading to disability in a hierarchy along this continuum [3].

The burden of disability among PLWH varies significantly temporally and spatially. The burden of disease has decreased globally, but some parts of the world, including some Asian countries, are experiencing an increase in deaths and disabilities due to HIV [4]. Among older PLWH, disability has significantly increased since 1990 in all regions of Asia [5]. It is important to understand the latest burden of disability among PLWH in Asia, with a goal to improve healthcare support services and quality of life among PLWH.

The proportion of PLWH older than 50 years has tripled globally since 2000, reaching 7.5 million in 2020, and has been rising significantly in both high‐ and middle‐ and low‐income countries [6]. The ageing population of PLWH experienced complex health issues, including earlier development of chronic comorbidities [7] associated with increased background chronic inflammation, as well as delayed healthcare access due to intersectional stigma arising from living with HIV, sexual orientation and ageing [8]. Physical, mental and socio‐behavioural factors all contribute to the multi‐dimensional nature of health and longevity in PLWH [9, 10, 11, 12]. In other words, impairments in these various domains of health bring functional impairment and disability to PLWH, while support and strategies that can potentially improve domains of health may reduce the level of disability [13].

The World Health Organization Disability Assessment Schedule (WHODAS) was developed for the cross‐cultural measurement of health status and functioning in major life domains [14], and has been applied in PLWH for various purposes, including characterizing the burden of disability, risk factors and impact of interventions [9, 10, 12, 15]. This is a well‐established tool to study the burden and determinants of disability in PLWH.

The prevalence and risk factors of disability among PLWH had been studied mainly in sub‐Saharan Africa, North America and Europe [1, 9, 10, 11], but the burden in Asian countries is poorly understood, nor are the determinants of disability well characterized. This study aimed to determine the prevalence of disability among PLWH living in Asia, as compared with matched controls living without HIV. We also aimed to determine the socio‐behavioural, physical and mental health determinants mediating disability among PLWH.

## 2 METHODS

### 2.1 Study design

We conducted a cross‐sectional case‐control study in five Asian cities, including Chongqing, Hong Kong, Kuala Lumpur, Singapore and Taipei, from March 2020 to November 2023. Individuals living with and without HIV were enrolled, and we collected demographic, socio‐behavioural and clinical data, and measured disability, frailty and parameters reflecting physical and mental health. We determined the prevalence of disability among PLWH, whether living with HIV was independently associated with disability and the variables associated with disability among PLWH. The study protocol was approved by the Joint Chinese University of Hong Kong‐New Territories East Cluster Clinical Research Ethics Committee, the institutional review board of University Malaya Medical Centre (UMMC), the Ethics Committee of Chongqing Public Health Medical Center, the Research Ethics Committee of National Taiwan University Hospital and the National Healthcare Group Domain Specific Review Board, Singapore.

### 2.2 Study population

PLWH were enrolled from HIV clinics in the five Asian cities. Inclusion criteria were a positive HIV antibody test, aged 35 years or above and Asian race. People who had acute medical problems requiring in‐hospital care and inability to provide informed consent were excluded from this study.

Age‐ (within ±5 years of age) and sex‐matched controls who tested negative for the HIV antibody test were enrolled from general medical clinics, infectious disease clinics, HIV pre‐exposure prophylaxis clinics and the community. In different cities, controls were enrolled in a 1:2 or 3 ratios compared with PLWH.

### 2.3 Outcome measures

The primary outcome was the presence of disability, as defined by short‐form WHODAS 2.0 (12‐item) score ≥2, which represents mild to moderate limitation in at least two, or moderate to severe limitation in at least one WHODAS item. These items represent an individual's functioning in six major life domains, including cognition (understanding and communication), mobility (moving and getting around), self‐care (personal hygiene and living alone), getting along (interacting with other people), life activities (carrying out responsibilities at home, work or school) and participation in society (engaging in community and recreational activities). This criterion was selected as it has been validated among PLWH in both high‐income and resource‐limited settings [9, 16], and it represents either moderate to severe impairment in functioning in one important life domain, or mild to moderate impairment in functioning at two or more life domains.

Secondary outcomes included a complex score of long‐form WHODAS 2.0 (36 item), impairment in each of its six functioning domains and frailty status. For complex scoring of WHODAS 2.0, item response theory‐based scoring was adopted, with each item response being treated separately and the summary score being generated by differentially weighting the items and levels of severity [14]. The complex score calculation followed the syntax for automatic computation provided by WHODAS 2.0 [17]. Each of the functioning domains was scored with a dichotomous scale, where the response scale for “mild,” “moderate,” “severe” and “extreme” for any item within the domain were all merged into a single positive coding, representing impairment [17]. Frailty was determined by the Fried frailty phenotype, with frailty defined by the presence of three or more abnormalities among the five items of unintentional weight loss, weak handgrip strength, exhaustion, slow gait speed and low physical activity [18].

### 2.4 Study procedures and data collection

Training was provided to research personnel involved in this study for all participating sites regarding the study procedures and definition of all parameters, to ensure the collection of data was standardized across all sites. All data were input by trained personnel into REDCap, an electronic data capture system, where all parameters were recorded in a standardized format.

We systematically collected socio‐demographic data, including date of birth, sex, marital status, employment status, smoking, alcohol use and substance use. Social support was measured by the revised version of the Lubben Social Network Scale [19]. We reviewed participants’ medical records to collect clinical, treatment and laboratory data. Blood pressure, body weight and height were measured, and the body mass index was calculated at the point of recruitment. Mental health was assessed by Patient Health Questionnaire‐9, Depression Anxiety Stress Scales (DASS‐21) and De Jong Gierveld Loneliness Scale [20]. Cognitive function was measured by the Montreal Cognitive Assessment test (MoCA) and the International HIV dementia scale. Details of the data collected are available in Supplementary Information.

### 2.5 Statistical analyses

Participants’ socio‐demographic and clinical characteristics and the outcome parameters were presented as number (proportions), mean ± standard deviation or median (lower quartile, upper quartile) as appropriate. Missing values (which were present in 0.1−2.8% of variables) were replaced with the mean of the available values for that variable. We compared categorical variables using chi‐square test, and continuous variables with Student's *t*‐test or Mann−Whitney U test, as appropriate.

We performed multivariable logistic regression analysis, adjusting for different variables: (1) demographics, including age, sex and site of enrolment; (2) socio‐behavioural variables, including marital status, smoking, substance use and social support; (3) physical health, including Charlson comorbidity index and body mass index; (4) mental health, including depression, anxiety, stress and loneliness; and (5) cognition, to determine association between living with HIV and primary and secondary outcomes. For the association with the WHODAS 2.0 complex score, we performed linear regression analysis adjusting for the same variables.

We also performed mediation analysis to assess the association and mediators between living with HIV and disability, as represented by the WHODAS 2.0 complex score. We performed path analysis to determine the role of multiple mediators in the direct and indirect effects of living with HIV on disability. PROCESS Model 4 was used, and overlapping effects among mediators were handled by estimating all mediators simultaneously in the outcome model, adjusting for covariation. This method isolates each mediator's unique indirect effect by accounting for shared variance with other mediators in the model. The mediators selected in the analysis included socio‐behavioural characteristics, physical health, mental health and cognition parameters, which were significantly different between PLWH and controls. The indirect effect of living with HIV on disability mediated by each of the parameters was calculated by multiplying the effect of living with HIV on that parameter by the effect of that parameter on disability, as expressed by unstandardized coefficients.

A multivariable logistic binary regression model was performed to determine variables independently associated with disability among PLWH, controlling for demographics, site of recruitment and variables with significant correlation with disability in univariate analyses.

All analyses were performed using SPSS Statistics v 29.0 (IBM Corp), with the two‐tailed significance level of *p*<0.05. For mediation analysis, the PROCESS macro in SPSS was used to test the multiple mediators in the association between living with HIV and disability [21].

## 3 RESULTS

### 3.1 Characteristics of the participants

During the study period, we enrolled 1004 PLWH and 416 age‐ and sex‐matched controls in the five cities in Asia. PLWH had a mean age of 53.6 ± 10.3 years, 84.4% were male and 72.2% had one or more comorbidities. Controls had a mean age of 53.2 ± 11.2 years, 84.1% were male and 66.8% had one or more comorbidities. The median duration of HIV diagnosis was 10.2 (4.6, 15.7) years, 51.0% had a past history of AIDS‐defining illnesses and 96.4% were receiving antiretroviral therapy (ART). Compared with controls, a lower proportion of PLWH were married, more of them had a history of smoking and substance use, and a higher Charlson Comorbidity Index. PLWH also had a lower body mass index, higher proportions of depression, anxiety and stress, a lower level of social support, a higher degree of loneliness and poorer performance on assessments of cognition. The characteristics of PLWH and controls are shown in Table 1 and Table S1.

**Table 1:** Socio‐demographic and clinical characteristics concerning physical and mental health of PLWH and age‐ and sex‐matched controls^a^

	All			Male sex			Female sex		
Variables	PLWH	Control	*p* value	PLWH	Control	*p* value	PLWH	Control	*p* value
Number	1004	416		847	350		157	66	
**Demographic characteristics**									
Site of recruitment			0.010			0.017			0.800
Hong Kong	300 (29.9%)	99 (23.8%)		258 (30.5%)	84 (24.0%)		42 (26.8%)	15 (22.7%)	
Kuala Lumpur	207 (20.6%)	67 (16.1%)		164 (19.4%)	52 (14.9%)		43 (27.4%)	15 (22.7%)	
Chongqing	200 (19.9%)	100 (24.0%)		134 (15.8%)	67 (19.1%)		66 (42.0%)	33 (50.0%)	
Taipei	200 (19.9%)	100 (24.0%)		197 (23.3%)	99 (28.3%)		3 (1.9%)	1 (1.5%)	
Singapore	97 (9.7%)	50 (12.0%)		94 (11.1%)	48 (13.7%)		3 (1.9%)	2 (3.0%)	
Male	847 (84.4%)	350 (84.1%)	0.914						
Age (years)	53.6 ± 10.3	53.2 ± 11.2	0.515	53.3 ± 10.5	52.4 ± 11.3	0.194	55.2 ± 9.4	57.5 ± 9.3	0.098
**Socio‐behavioural factors**									
Married	343 (34.2%)	295 (70.9%)	<0.001	252 (29.8%)	246 (70.3%)	<0.001	91 (58.0%)	49 (74.2%)	0.022
No employment	325 (32.4%)	124 (29.8%)	0.339	267 (31.6%)	96 (27.4%)	0.157	58 (36.9%)	28 (42.4%)	0.433
Ever smoker	473 (47.1%)	145 (34.9%)	<0.001	449 (53.0%)	142 (40.6%)	<0.001	24 (15.3%)	3 (4.5%)	0.025
Current smoker	253 (25.2%)	59 (14.2%)	<0.001	237 (28.0%)	57 (16.3%)	<0.001	16 (10.2%)	2 (2.0%)	0.073
Alcohol use more than once per month	174 (17.4%)	82 (19.7%)	0.299	160 (19.0%)	82 (23.4%)	0.080	14 (8.9%)	0 (0%)	0.012
Substance use	193 (19.2%)	20 (4.8%)	<0.001	187 (22.1%)	19 (5.4%)	<0.001	6 (3.8%)	1 (1.5%)	0.677
Lubben social network scale	12.6 ± 6.2	15.9 ± 5.7	<0.001	12.7 ± 6.3	15.7 ± 5.9	<0.001	12.3 ± 5.8	17.1 ± 4.5	<0.001
**Physical health**									
Charlson Comorbidity Index	1 (0, 2)	1 (0, 2)	0.016	1 (0, 2)	1 (0, 2)	0.006	2 (1, 3)	2 (1, 2)	0.858
Presence of any comorbidity	725 (72.2%)	278 (66.8%)	0.043	598 (70.6%)	221 (63.1%)	0.012	127 (80.9%)	57 (86.4%)	0.326
Comorbidities									
Hypertension	300 (29.9%)	98 (23.6%)	0.016	262 (30.9%)	82 (23.4%)	0.009	38 (24.2%)	16 (24.2%)	0.995
Diabetes	199 (19.8%)	66 (15.9%)	0.082	173 (20.4%)	54 (15.4%)	0.045	26 (16.6%)	12 (18.2%)	0.769
Liver disease	105 (10.5%)	21 (5.0%)	0.001	83 (9.8%)	15 (4.3%)	0.002	22 (14.0%)	6 (9.1%)	0.311
Cardiovascular disease	103 (10.3%)	19 (4.6%)	<0.001	76 (9.0%)	15 (4.3%)	0.005	27 (17.2%)	4 (6.1%)	0.028
Osteoporosis	56 (5.6%)	2 (0.5%)	<0.001	29 (3.4%)	0 (0%)	<0.001	27 (17.2%)	2 (3.0%)	0.004
Chronic pulmonary diseases	26 (2.6%)	12 (2.9%)	0.754	23 (2.7%)	12 (3.4%)	0.505	3 (1.9%)	0 (0%)	0.557
Chronic kidney disease	24 (2.4%)	4 (1.0%)	0.078	20 (2.4%)	2 (0.6%)	0.036	4 (2.5%)	2 (3.0%)	1.000
Cancer	24 (2.4%)	7 (1.7%)	0.406	17 (2.0%)	6 (1.7%)	0.737	7 (4.5%)	1 (1.5%)	0.441
Body weight (kg)	67.8 ± 13.9	69.3 ± 13.4	0.050	69.4 ± 13.4	70.7 ± 13.1	0.105	59.2 ± 13.7	61.9 ± 12.8	0.159
Body mass index (kg/m^2^)	24.2 ± 4.3	24.7 ± 4.0	0.042	24.3 ± 4.2	24.8 ± 3.9	0.100	23.4 ± 4.8	24.3 ± 4.3	0.197
Systolic blood pressure (mmHg)	130.9 ± 17.9	130.1 ± 16.1	0.403	131.4 ± 17.8	130.2 ± 15.8	0.228	128.1 ± 18.0	129.8 ± 17.7	0.543
Diastolic blood pressure (mmHg)	82.4 ± 11.4	82.3 ± 10.2	0.892	82.6 ± 11.3	82.4 ± 10.1	0.757	81.1 ± 11.5	81.7 ± 10.9	0.725
**Mental health**									
PHQ‐9 total score	3 (0, 6)	1 (0, 4)	<0.001	2 (0, 6)	1 (0, 4)	<0.001	4 (1, 7)	1 (0, 4)	<0.001
Moderate or severe depression (PHQ‐9 ≥10)	82 (8.2%)	13 (3.1%)	<0.001	60 (7.1%)	12 (3.4%)	0.016	22 (14.0%)	1 (1.5%)	0.005
DASS anxiety subscale	1 (0, 3)	1 (0, 2)	<0.001	1 (0, 3)	1 (0, 2)	<0.001	2 (1, 4)	1 (0, 2)	<0.001
Anxiety (DASS anxiety subscale ≥8)	66 (6.6%)	11 (2.6%)	0.003	55 (6.5%)	8 (2.3%)	0.003	11 (7.0%)	3 (4.5%)	0.763
DASS stress subscale	2 (0, 5)	1 (0, 3)	<0.001	2 (0, 5)	1 (0, 3)	<0.001	3 (0, 6)	1 (0, 4)	0.019
Stress (DASS stress subscale ≥15)	110 (11.0%)	24 (5.8%)	0.002	92 (10.9%)	20 (5.7%)	0.005	18 (11.5%)	4 (6.1%)	0.217
Loneliness scale	3.08 ± 1.81	2.79 ± 1.66	0.004	3.09 ± 1.82	2.82 ± 1.71	0.019	3.03 ± 1.78	2.62 ± 1.33	0.060
**Cognition**									
International HIV dementia scale	9.49 ± 1.91	9.97 ± 1.65	<0.001	9.60 ± 1.89	10.06 ± 1.64	<0.001	8.88 ± 1.90	9.53 ± 1.64	0.015
Montreal Cognitive Assessment test	26 (22, 28)	27 (24, 29)	<0.001	26 (23, 29)	27 (25, 29)	0.004	22 (17, 25)	25 (18, 27)	0.025
**HIV‐related variables**									
Duration of HIV (years)	10.2 (4.6, 15.7)			10.6 (5.9, 15.8)			6.6 (1.0, 15.0)		
Past history of AIDS‐defining illness	508 (51.0%)			416 (49.1%)			92 (58.6%)		
Current CD4 count (cells/mm^3^)	540 (346, 720)			537 (353, 719)			505 (254, 682)		
Current CD8 count (cells/mm^3^)	685 (467, 940)			711 (501, 977)			616 (403, 813)		
Current CD4:CD8 ratio	0.75 (0.49, 1.12)			0.72 (0.48, 1.04)			0.75 (0.47, 1.08)		
Current viral load (copies per ml)	20 (20, 34)			20 (20, 34)			34 (20, 50)		
HIV RNA <50 copies per ml	842 (84.5%)			723 (85.4%)			119 (75.8%)		
Receiving ART	968 (96.4%)			819 (96.7%)			149 (94.9%)		
Current ART regimen									
Nucleoside reverse transcriptase inhibitor	949 (94.5%)			806 (98.4%)			143 (96.0%)		
Non‐nucleoside reverse transcriptase inhibitor	363 (36.2%)			290 (35.4%)			73 (49.0%)		
Protease inhibitor	90 (9.0%)			75 (9.2%)			15 (10.1%)		
Integrase strand transfer inhibitor	536 (53.4%)			469 (57.3%)			67 (45.0%)		

Abbreviations: ART, antiretroviral therapy; DASS, Depression Anxiety Stress Scales; PHQ‐9, Patient Health Questionnaire ‐9.

^a^ Data are presented as number (proportion), mean ± standard deviation or median (lower quartile, upper quartile), as appropriate.

### 3.2 Burden of disability and frailty among PLWH and controls

Table 2 shows the outcome parameters for PLWH and controls. The prevalence of disability was 50.9% among PLWH and 40.6% among controls (*p*<0.001). PLWH had a significantly higher WHODAS 2.0 complex score, and significantly more PLWH had impairments in all of the six domains of disability, namely cognition, mobility, self‐care, getting along with others, life activity and social participation. Likewise, significantly higher proportions of PLWH had frail (6.6% vs. 1.7%) and pre‐frail status (54.0% vs. 45.9%), and had a deficit in two of the domains of the frailty phenotype, namely exhaustion and handgrip strength.

**Table 2:** The presence of disability and frailty according to sex and HIV status

	All			Male sex			Female sex		
Outcomes	PLWH	Control	*p* value	PLWH	Control	*p* value	PLWH	Control	*p* value
**Number**	1004	416		847	350		157	66	
Disability (WHODAS score ≥2)	511 (50.9%)	169 (40.6%)	<0.001	421 (49.7%)	140 (40.0%)	0.002	90 (57.3%)	29 (43.9%)	0.067
WHODAS complex score (without work)	5.43 (1.09, 11.96)	3.26 (0.00, 8.70)	<0.001	4.35 (1.09, 11.96)	3.26 (0.00, 8.70)	<0.001	7.61 (3.26, 17.39)	3.80 (1.09, 10.87)	<0.001
Impairments in									
Cognition	457 (46.3%)	168 (40.4%)	0.043	376 (45.1%)	146 (41.7%)	0.287	81 (52.6%)	22 (33.3%)	0.009
Mobility	359 (36.3%)	116 (27.9%)	0.002	297 (35.6%)	91 (26.0%)	0.001	62 (40.3%)	25 (37.9%)	0.741
Self‐care	116 (11.7%)	29 (7.0%)	0.007	90 (10.8%)	22 (6.3%)	0.016	26 (16.9%)	7 (10.6%)	0.232
Getting along	380 (38.5%)	126 (30.3%)	0.004	322 (38.6%)	110 (31.4%)	0.019	58 (37.7%)	16 (24.2%)	0.054
Life activity	254 (25.7%)	74 (17.8%)	0.001	211 (25.3%)	60 (17.1%)	0.002	43 (27.9%)	14 (21.2%)	0.298
Participation	714 (72.3%)	247 (59.4%)	<0.001	586 (70.3%)	205 (58.6%)	<0.001	128 (83.1%)	42 (63.6%)	0.002
Frailty status			<0.001			<0.001			0.040
Robust	396 (39.4%)	218 (52.4%)		331 (39.1%)	186 (53.1%)		65 (41.4%)	32 (48.5%)	
Pre‐frail	542 (54.0%)	191 (45.9%)		469 (55.4%)	158 (45.1%)		73 (46.5%)	33 (50.0%)	
Frail	66 (6.6%)	7 (1.7%)		47 (5.5%)	6 (1.7%)		19 (12.1%)	1 (1.5%)	
Frailty phenotype domains									
Exhaustion	223 (22.2%)	70 (16.8%)	0.022	181 (21.4%)	59 (16.9%)	0.076	42 (26.8%)	11 (16.7%)	0.106
Unintentional weight loss	105 (10.5%)	35 (8.4%)	0.239	80 (9.4%)	31 (8.9%)	0.750	25 (15.9%)	4 (6.1%)	0.046
Low physical activity	215 (21.4%)	72 (17.3%)	0.079	157 (20.7%)	57 (16.3%)	0.082	40 (25.5%)	15 (22.7%)	0.664
Weak handgrip	349 (34.8%)	71 (17.1%)	<0.001	308 (36.4%)	62 (17.7%)	<0.001	41 (26.1%)	9 (13.6%)	0.041
Slow gait speed	38 (3.8%)	8 (1.9%)	0.071	26 (3.1%)	7 (2.0%)	0.304	12 (7.6%)	1 (1.5%)	0.062

Abbreviation: WHODAS, World Health Organization Disability Assessment Schedule.

### 3.3 Association of living with HIV with disability and frailty

The presence of disability and impairments in mobility, self‐care, getting along, life activity and participation correlated with living with HIV after adjusting for demographic characteristics. These associations also remained significant after adjusting for demographic characteristics and physical health parameters. Living with HIV was no longer significantly correlated with these parameters (with the exception of participation) after adjusting for demographic characteristics and socio‐behavioural variables or mental health parameters. Living with HIV significantly correlated with disability, and impairments in getting along, life activity and participation, after adjusting for cognition. Living with HIV significantly correlated with the presence of frailty after adjusting for all socio‐behavioural, physical health, mental health and cognition parameters. The correlations between living with HIV and disability and frailty are shown in Table 3.

**Table 3:** The correlation between HIV status and disability and frailty, adjusted by socio‐demographic and physical and mental health‐related variables

	Unadjusted model	Adjusted for age, sex, site of recruitment	Adjusted Model 1 (demographic and socio‐behavioural)	Adjusted Model 2 (demographic and physical health)	Adjusted Model 3 (demographic and mental health)	Adjusted Model 4 (demographic and cognition)
	**Odds ratio, *p* value**	**Adjusted odds ratio, *p* value**				
Disability (WHODAS 2.0 ≥2)	**1.515** **(1.202, 1.910),** ***p*<0.001**	**1.441** **(1.136, 1.828),** ***p* = 0.003**	1.108 (0.851, 1.444), *p* = 0.445	**1.397** **(1.099, 1.776),** ***p* = 0.006**	1.244 (0.962, 1.608), *p* = 0.096	**1.330** **(1.044, 1.694),** ***p* = 0.021**
Impairment in:						
Cognition	**1.270** **(1.007, 1.603),** ***p* = 0.043**	1.158 (0.906, 1.480), *p* = 0.241	0.991 (0.752, 1.307), *p* = 0.951	1.121 (0.876, 1.436), *p* = 0.364	1.018 (0.788, 1.315), *p* = 0.893	1.059 (0.825, 1.360). *p* = 0.652
Mobility	**1.476** **(1.149, 1.896),** ***p* = 0.002**	**1.379** **(1.062, 1.790),** ***p* = 0.016**	1.051 (0.785, 1.406), *p* = 0.738	**1.341** **(1.029, 1.748),** ***p* = 0.030**	1.214 (0.927, 1.591), *p* = 0.159	1.227 (0.941, 1.601). *p* = 0.131
Self‐care	**1.775** **(1.162, 2.713),** ***p* = 0.008**	**1.686** **(1.097, 2.593),** ***p* = 0.017**	1.268 (0.808, 1.990), *p* = 0.302	**1.684** **(1.091, 2.598),** ***p* = 0.019**	1.333 (0.854, 2.081), *p* = 0.206	1.477 (0.954, 2.285), *p* = 0.080
Getting along	**1.438** **(1.126, 1.838),** **0.004**	**1.397** **(1.087, 1.796),** ***p* = 0.009**	1.133 (0.868, 1.478), *p* = 0.358	**1.375** **(1.068, 1.769),** ***p* = 0.013**	1.208 (0.926, 1.575), *p* = 0.163	**1.332** **(1.033, 1.717)**. ***p* = 0.027**
Life activity	**1.599** **(1.198, 2.135),** ***p* = 0.001**	**1.492** **(1.107, 2.011),** ***p* = 0.009**	1.244 (0.912, 1.697), 0.168	**1.462** **(1.083, 1.973),** ***p* = 0.013**	1.252 (0.915, 1.714), *p* = 0.160	**1.359** **(1.004, 1.841),** ***p* = 0.047**
Participation	**1.783** **(1.402, 2.267),** ***p*<0.001**	**1.803** **(1.409, 2.308),** ***p*<0.001**	**1.598** **(1.239, 2.061),** ***p*<0.001**	**1.763** **(1.376, 2.259),** ***p*<0.001**	**1.672** **(1.289, 2.168),** ***p*<0.001**	**1.741** **(1.357, 2.233),** ***p*<0.001**
Frailty	**4.111** **(1.870, 9.038),** ***p*<0.001**	**4.273** **(1.933, 9.449),** ***p*<0.001**	**3.857** **(1.621, 7.130),** ***p* = 0.001**	**4.177** **(1.881, 9.273),** ***p*<0.001**	**3.725** **(1.670, 8.307),** ***p* = 0.001**	**3.253** **(1.453, 7.281),** ***p* = 0.004**
	**Unstandardized coefficient B**					
WHODAS 2.0 Complex score	**3.386, *p*<0.001**	**3.231, *p*<0.001**	**1.708, *p* = 0.010**	**3.010, *p*<0.001**	**1.867, *p* = 0.001**	**2.462, *p*<0.001**

*Note*: Variables included in the different models:

Model 1: age, sex, site, marital status, smoking, substance use, Lubben social network.

Model 2: age, sex, site, Charlson comorbidity index, body mass index.

Model 3: age, sex, site, depression (PHQ‐9 ≥10), anxiety (DASS anxiety subscale ≥8), stress (DASS stress subscale ≥15), loneliness scale.

Model 4: age, sex, site, International HIV dementia scale, Montreal Cognitive Assessment test.

Correlations that are considered significant (*p* <0.05) are in bold type.

Abbreviations: DASS, Depression Anxiety Stress Scales; PHQ‐9, Patient Health Questionnaire ‐9; WHODAS, World Health Organization Disability Assessment Schedule.

**Figure 1:**
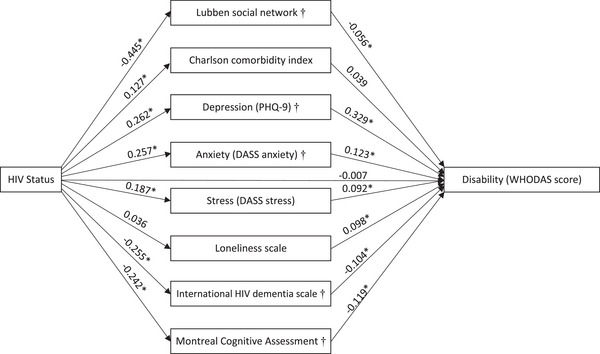
Path analysis of World Health Organization Disability Assessment Schedule (WHODAS) score, standardized coefficient is presented in path. The model includes age, sex, site, marital status, smoking, substance use and BMI. *p<0.05, † *p*<0.05 for indirect effect of HIV infection on disability. Abbreviations: DASS, Depression Anxiety Stress Scales; PHQ‐9, Patient Health Questionnaire‐9.

Results of path analysis showed that living with HIV did not have a significant direct effect on disability. On the other hand, living with HIV had a significant effect on four mediators, namely social isolation, burden of comorbidities, mental health, including depression, anxiety and stress, as well as cognition, with standardized coefficients ranging from −0.445 to 0.262 (all *p*‐values <0.05). Moreover, living with HIV had an indirect association with disability through three of these mediators, including social isolation, mental health, that is depression and anxiety, and measurements of cognition. The results of path analysis are shown in Figure 1. The strongest indirect effect of living with HIV on disability was mediated by depression (β = 0.982, *p*<0.05), which accounted for 39.71% of the total effect of living with HIV on disability, followed by anxiety (β = 0.361, *p*<0.05), which accounted for 14.60% of the total effect, and cognitive function as measured by MoCA (β = 0.330, *p*<0.05), which accounted for 13.34% of the total effect (Table S2).

### 3.4 Determinants of disability among PLWH

We compared the demographic, socio‐behavioural and health‐related variables between PLWH with and those without disability (Table 4). PLWH without employment, higher burden of comorbidities, worse mental health, poorer cognitive function and poorer social support were associated with a higher risk of disability. HIV‐related variables were not associated with the presence of disability. Multivariable analysis showed that disability among PLWH was independently associated with site of recruitment, cardiovascular disease, depression, anxiety, stress, MoCA and loneliness.

**Table 4:** Demographic, socio‐behavioural and health‐related variables associated with disability among PLWH

	Disability *N* = 511	No disability *N* = 493	*p* value	Odds ratio (95% confidence interval)	*p* value	Adjusted odds ratio (95% confidence interval)	*p* value
**Demographic characteristics**							
Site							
Hong Kong	167 (32.7%)	133 (27.0%)		Reference			
Chongqing	98 (19.2%)	102 (20.7%)	<0.001	0.765 (0.534, 1.095)	0.144	0.531 (0.329, 0.859)	0.010
Kuala Lumpur	129 (25.2%)	78 (15.8%)		1.317 (0.917, 1.891)	0.136	1.323 (0.882, 1.986)	0.176
Singapore	20 (3.9%)	77 (15.6%)		0.207 (0.120, 0.356)	<0.001	0.246 (0.131, 0.460)	<0.001
Taipei	97 (19.0%)	103 (20.9%)		0.750 (0.524, 1.074)	0.116	1.075 (0.697, 1.658)	0.743
Male	421 (82.4%)	426 (86.4%)	0.079	1.359 (0.964, 1.917)	0.080		
Age (years)	54.1 ± 10.8	53.1 ± 9.8	0.146	1.009 (0.997, 1.021)	0.147		
**Socio‐behavioural factors**							
Married	168 (32.9%)	175 (35.5%)	0.381	0.890 (0.686, 1.155)	0.382		
No employment	184 (36.1%)	141 (28.6%)	0.011	1.409 (1.080, 1.839)	0.012		
Current smoker	130 (25.4%)	123 (24.9%)	0.858	1.026 (0.772, 1.365)	0.858		
Ever smoker	249 (48.7%)	224 (45.4%)	0.296	1.141 (0.891, 1.463)	0.296		
Alcohol use more than once per month	84 (16.4%)	90 (18.3%)	0.438	0.879 (0.633, 1.219)	0.438		
Substance use (ever)	90 (17.6%)	103 (20.9%)	0.187	0.809 (0.591, 1.109)	0.188		
Lubben social network scale	11.56 ± 6.02	13.73 ± 6.17	<0.001	0.943 (0.924, 0.963)	<0.001		
**Physical health**							
Comorbidities							
Hypertension	168 (32.9%)	132 (26.8%)	0.035	1.340 (1.021, 1.758)	0.035		
Diabetes	111 (21.7%)	88 (17.8%)	0.124	1.277 (0.935, 1.745)	0.124		
Liver diseases	55 (10.8%)	50 (10.1%)	0.748	1.069 (0.713, 1.602)	0.748		
Cardiovascular diseases	77 (15.1%)	26 (5.3%)	<0.001	3.187 (2.005, 5.065)	<0.001	3.719 (2.144, 6.451)	<0.001
Osteoporosis	30 (5.9%)	26 (5.3%)	0.680	1.120 (0.653, 1.923)	0.680		
Chronic pulmonary diseases	17 (3.3%)	9 (1.8%)	0.134	1.851 (0.817, 4.192)	0.140		
Chronic kidney diseases	19 (3.7%)	5 (1.0%)	0.005	3.769 (1.396, 10.175)	0.009		
Cancer	13 (2.5%)	11 (2.2%)	0.746	1.144 (0.508, 2.578)	0.746		
Body weight (kg)	67.9 ± 14.7	67.7 ± 13.0	0.807	1.001 (0.992, 1.010)	0.807		
Body mass index (kg/m^2^)	24.3 ± 4.6	24.1 ± 3.9	0.466	1.011 (0.982, 1.041)	0.467		
Systolic blood pressure (mmHg)	130.4 ± 19.7	132.0 ± 18.6	0.190	0.993 (0.986, 1.000)	0.042		
Diastolic blood pressure (mmHg)	82.9 ± 12.2	82.2 ± 12.5	0.350	1.003 (0.992, 1.014)	0.619		
**Mental health**							
Moderate or severe depression (PHQ‐9 ≥10)	76 (14.9%)	6 (1.2%)	<0.001	1.316 (1.258, 1.376)	<0.001	4.635 (1.726, 12.442)	0.002
Anxiety (DASS anxiety subscale ≥8)	63 (12.3%)	3 (0.6%)	<0.001	22.969 (7.162, 73.657)	<0.001	5.070 (1.389, 18.503)	0.014
Stress (DASS stress subscale ≥15)	101 (19.8%)	9 (1.8%)	<0.001	13.248 (6.616, 26.528)	<0.001	5.471 (2.393, 12.508)	<0.001
Loneliness scale	3.72 ± 1.73	2.42 ± 1.65	<0.001	1.550 (1.432, 1.677)	<0.001	1.372 (1.256 (1.498)	<0.001
**Cognition**							
International HIV dementia scale	9.25 ± 2.02	9.73 ± 1.75	<0.001	0.876 (0.819, 0.937)	<0.001		
Montreal Cognitive Assessment test	25 (22, 28)	27 (23, 29)	<0.001	0.956 (0.932, 0.981)	<0.001	0.939 (0.904, 0.974)	<0.001
**HIV‐related variables**							
Duration of HIV (years)	10.9 (5.1, 16.3)	9.8 (4.2, 15.3)	0.135	1.015 (0.997, 1.032)	0.097		
History of AIDS‐defining illness	267 (52.3%)	238 (48.9%)	0.286	1.145 (0.893, 1.468)	0.286		
Current CD4 count (cells/mm^3^)	525 (336, 712)	539 (340, 714)	0.369	1.000 (0.999, 1.000)	0.337		
Current CD8 count (cells/mm^3^)	685 (473, 942)	714 (484, 958)	0.336	1.000 (1.000, 1.000)	0.503		
Current CD4: CD8 ratio	0.72 (0.47, 1.08)	0.74 (0.49, 1.04)	0.585	0.976 (0.757, 1.259)	0.853		
Current viral load	20 (20, 34)	20 (20, 34)	0.004	1.000 (1.000, 1.000)	0.558		
HIV RNA <50 copies per ml	421 (82.4%)	421 (85.4%)	0.075	0.730 (0.516, 1.033)	0.076		
Receiving ART	489 (95.7%)	479 (97.2%)	0.212	0.650 (0.329, 1.285)	0.215		
Current ART regimen							
Non‐nucleoside reverse transcriptase inhibitor	169 (34.6%)	194 (40.5%)	0.038	0.762 (0.588, 0.986)	0.039		
Protease inhibitor	52 (10.6%)	38 (7.9%)	0.171	1.356 (0.875, 2.102)	0.172		
Integrase strand transfer inhibitor	283 (57.9%)	253 (52.8%)	0.197	1.177 (0.919, 1.509)	0.197		

Abbreviations: ART, antiretroviral therapy; DASS, Depression Anxiety Stress Scales; PHQ‐9, Patient Health Questionnaire ‐9.

## 4 DISCUSSION

In this multi‐country cross‐sectional study in Asia, PLWH had a higher burden of disability as compared with age‐ and sex‐matched controls living without HIV. PLWH had more significant impairments in functioning in all life domains. Living with HIV indirectly impacted disability through worse mental health, poorer cognitive function and social isolation.

Studies from North America and Europe showed that PLWH had a two‐ to three‐fold higher burden of disability than the general population [22, 23, 24]. Among people with various comorbidities, PLWH had a higher burden of disability with more disability‐adjusted life‐years [23]. In most studies from low‐ or middle‐income countries, living with HIV was associated with disability in men, women and adolescents [25, 26].

Our study provides data showing that the burden of disability among PLWH residing in various Asian countries is higher than age‐ and sex‐matched controls. The major strengths of our study include a prospective study design collecting standardized parameters involving different health‐related domains across study sites, involving a large sample of PLWH and matched controls living without HIV. Participants in this study came from both high‐ and low‐ or middle‐income countries, with a range of socio‐demographic backgrounds, receiving different ART regimens, reflecting real‐world settings in Asia, where different countries share similar cultural and social characteristics while maintaining their uniqueness in socio‐demographic composition and health system structures.

We showed that significantly higher proportions of PLWH had impairment in all domains of functioning as compared with age‐ and sex‐matched controls living without HIV. This observation highlights the importance of measuring the multi‐dimensional facets of disability among PLWH, following a bio‐psycho‐social framework in clinical and community settings [9, 27]. Likewise, frailty is increasingly recognized as a stage along the continuum from robustness to disability [3], and should also be recognized as a multi‐dimensional condition [28]. Incorporation of monitoring for the development of multi‐dimensional frailty in the routine care of older PLWH would be beneficial in identifying those at risk of future disability and implementing suitable interventions.

Similar to other studies, among the six domains of functioning, the greatest difference in impairment between PLWH and controls was observed in “participation” [9, 29]. This domain concerns participation in society and the impact of health problems on individuals and their families. Social participation and engagement are one of the major research priorities in HIV and ageing, and innovative strategies, such as digital technology, should be explored to positively enhance social interactions [30].

In this study, we have investigated the mediating factors contributing to a higher burden of disability among PLWH. From multivariate logistic regression models, living with HIV was no longer associated with various dimensions of disability after adjusting for socio‐behavioural characteristics, mental health parameters and cognition. Similarly, from path analysis, we observed that living with HIV exerted an indirect effect on disability through social isolation, mental health and cognition. These findings suggest that PLWH had increased vulnerability to disability predominantly due to higher prevalence of associated conditions, including worse mental health and poorer cognitive function.

Among PLWH, social isolation contributes to poor physical and mental health outcomes as well as mortality [31]. Importantly, different dimensions of social support, including emotional, informational and positive social interaction, provide benefits to PLWH in reducing stigma and improving mental health [32]. Social isolation is seldom assessed in the clinical care of PLWH, and future research should focus on interventions that are effective in preventing social isolation and strengthening multi‐dimensional social support for PLWH with a goal to prevent disability.

Mental health disorders, including depression, anxiety and post‐traumatic stress disorder, influence physical health and disability in PLWH, as well as the severity of disability [10, 12, 33]. The relationship between mental health and disability is bi‐directional. While mood disorder is known to contribute to disability among PLWH, disability raises the risk of future development as well as delayed diagnosis and treatment of symptomatic depression among PLWH [34, 35].

Importantly, among PLWH, mood disorders and cognitive impairment appeared to cluster as a distinct biopsychosocial phenotype, and individuals with such a phenotype had the worst degree of impairment in daily functioning [36]. The relationship between mood disorder and cognitive impairment is also connected by stigma. Stigma exerts direct effects on both mood and cognitive performance in PLWH, as well as indirectly impacting impairments in daily function and engagement in social participation via these effects [37].

While HIV‐related factors, such as late HIV diagnosis and duration while living with HIV, have been found to be associated with disability in other studies [9, 10], in our study, none of the HIV‐related variables was shown to correlate with risk of disability. This may reflect the different aetiologies of advanced HIV disease and differences in recovery trajectory from advanced HIV disease in different geographic regions. In terms of physical health, living with HIV was shown to have a direct impact on the burden of comorbidities. Cardiovascular disease was a significant variable associated with disability among PLWH in our cohort. These observations support the importance of early diagnosis and intervention to prevent and treat age‐related comorbidities in PLWH, such as preventing cardiovascular diseases [38].

PLWH from the five cities involved in this study had different risks of disability, with PLWH from Chongqing and Singapore having a lower risk, while those from the other three cities having comparable risks (Table 4). This could be explained by variation in host characteristics, which were not fully adjusted in the model, but may also reflect cultural differences in the perception of disability, differences in healthcare infrastructure and factors related to environmental support that may have an impact on the functional ability of participants in different cities.

Our study had several limitations. The cross‐sectional nature of the study is inadequate to address disability as a dynamic process over time, which characterizes periods of wellness and impairment over an individual's life course [13]. Moreover, our study was only able to identify association, but not causality, between HIV acquisition and other factors and disability. Future longitudinal observational studies should be performed to determine factors and processes associated with fluctuation in disability and to identify tools to predict future development of disability. Moreover, the study period had started soon after the COVID‐19 pandemic; thus, the impact of the pandemic and measures of infection prevention may have affected the participants’ daily activities and mental health. There is currently no consensus regarding the optimal age to start screening of disability among PLWH, we have enrolled participants aged 35 years or above as PLWH in contemporary cohorts generally have a life expectancy >70 years of age [39], and for the majority of PLWH, age of 35 years approximates middle age, which is the time when functional ability starts to decline according to WHO life course model. We have also included the Asian race exclusively due to a lack of data in this population. These criteria may, however, limit the generalizability of our results to younger PLWH from other races. We have not collected data on participants’ gender and their history of ART regimens, and were unable to analyse the impact of gender and previous exposure to antiretroviral drugs on disability and associated factors, such a cognitive function.

At the moment, prevention and management of disability are not included in most national strategies in the control and treatment of HIV [40]. Disability should be advocated as a focus of national HIV treatment programmes, so that PLWH with disability will have their rights protected and have access to affordable care, including rehabilitation and other support services; healthcare providers will receive training to raise their awareness of the needs of PLWH with disability [1, 40]. Routine screening for disability, by short‐form WHODAS 2.0 or other locally validated tools, and measurement of intrinsic capacity should be included as part of HIV care. Screening and intervention for mental health and cognition should be part of routine care, just as the provision of social support services in the care of PLWH with disability [41]. Healthcare providers should be equipped with available resources in the healthcare system and community to alleviate the negative impact of disability on the daily functioning of PLWH.

## 5 CONCLUSIONS

This multi‐country study in Asia has shown that PLWH had a higher burden of disability as compared with age‐ and sex‐matched controls living without HIV. Living with HIV exerted an indirect effect on disability via social isolation, mental health disorders and cognitive impairment. Disability should be a focus of HIV care services with a goal to prevent the development of disability among PLWH and to provide accessible and equitable care to those with disability.

## COMPETING INTERESTS

GL has received a research grant from Gilead Sciences and MSD. The other authors do not have any declarations of conflict of interest.

## AUTHOR CONTRIBUTIONS

GL, YC, C‐CH, PLW, CSW and RR have access to the data in their corresponding study sites, and take responsibility for the integrity and accuracy of the data, and were responsible for the study concept and design. GL, YC, C‐CH, PLW, CSW, XX, CC, GL, VW and RR were responsible for the acquisition of data. GL, JL and TK were responsible for data analysis. GL drafted the manuscript. All authors had a significant contribution to the intellectual content of the manuscript and approved its final version.

## FUNDING

This work is supported by a research grant from Gilead Sciences (IN‐HK‐985‐5721) and a donation fund from GSK, both granted to GL.

## Supporting information



Supporting Information

## Data Availability

Data involved in this study can be shared on request, subject to approval by the respective institutional review boards.
