# Supplementary material for: Cognitive and mental health significantly contribute to disability in people ageing with HIV in Asia: an observational case‐control study
Source: J Int AIDS Soc. 2025 Oct 28;28(11):e70052. doi: 10.1002/jia2.70052 (PMC12568376; doi:10.1002/jia2.70052)
Supplement: Supplementary file 1 — Supporting Information [file JIA2-28-e70052-s001.docx]

**Supplementary methodology**

Study procedures and data collection

Training was provided to research personnel involved in this study for all participating sites regarding the study procedures and definition of all parameters, to ensure the collection of data was standardized across all sites. All data was input by trained personnel into REDCap, an electronic data capture system, where all parameters were recorded in a standardized format.

We systematically collected socio-demographic data, including date of birth, sex, marital status, employment status, smoking, alcohol use and substance use. Social support was measured by the revised version of Lubben Social Network Scale (LSNS-R) (1). This is a 12-item scale measuring size, closeness and frequency of contact with family, friends and neighbours, with a score ranging from 0 to 60, and higher scores indicating a greater level of social support and lower risk for isolation.

We reviewed participants’ medical records to collect clinical data for underlying comorbidities to determine the Charlson comorbidity index, year of HIV diagnosis, history of AIDS-defining illness, the latest HIV RNA level, CD4 count, and ART. Blood pressure and body weight and height were measured, and the body mass index was calculated at the point of recruitment.

Mental health was assessed by Patient Health Questionnaire-9 (PHQ-9) and Depression Anxiety Stress Scales (DASS-21). PHQ-9 scores range from 0-27, with moderate to severe degree of depression defined by PHQ-9 score ≥10 (2). DASS-21 scores range from 0-42 for each domain of stress, anxiety and depression. The presence of anxiety and stress were defined by anxiety subscale score ≥8 and stress subscale score ≥15 respectively (3). Loneliness was measured by the De Jong Gierveld Loneliness Scale, which is a measure of overall, emotional and social loneliness, with score ranging from 0 to 6, and a higher score indicating more severe loneliness (4).

Cognitive function was measured by the Montreal Cognitive Assessment test (MoCA) and the International HIV dementia scale (IHDS). MoCA was performed using the relevant language version for the different study sites. MoCA scores range from 0-30, while IHDS range from 0-12, both with higher scores indicating better cognitive function. These two tests were selected based on their complementary nature, with MoCA testing short-term memory, visuospatial abilities, executive functions, attention, concentration and language, while IHDS assessing motor speed and psychomotor speed (5).

The World Health Organization Disability Assessment Schedule (WHODAS) 2.0 was adopted to measure disability. Each participant rated each question on a 5-point Likert scale (range 0-4). The short-form WHODAS (12-item) presented a score ranging 0-48, with higher scores indicating greater disability. In the long-form WHODAS (36-item), the level of functioning in six domains of life (cognition, mobility, self-care, getting along, life activities and participation) were calculated according to “Measuring Health and Disability Manual for WHO Disability Assessment Schedule WHODAS 2.0”, and the summary score in complex scoring was converted into a metric ranging from 0 to 100, with 0 indicating no disability and 100 full disability (6).

References:

1. Perloff E, DeRoy AK, Brooks BL, Price-Kelly J, Alfred C, Cecchetti A, et al., editors. Refinements to the Lubben Social Network Scale: The LSNS-R2002.

2. Kroenke K, Spitzer RL, Williams JB. The PHQ-9: validity of a brief depression severity measure. J Gen Intern Med. 2001;16(9):606-13.

3. Lovibond SH, Lovibond PF, Psychology Foundation of A. Manual for the depression anxiety stress scales. 2nd ed ed. Sydney, N.S.W.: Psychology Foundation of Australia; 1995.

4. Gierveld JDJ, Tilburg TV. A 6-Item Scale for Overall, Emotional, and Social Loneliness:Confirmatory Tests on Survey Data. Research on Aging. 2006;28(5):582-98.

5. Chartier M, Crouch PC, Tullis V, Catella S, Frawley E, Filanosky C, et al. The Montreal Cognitive Assessment: A Pilot Study of a Brief Screening Tool for Mild and Moderate Cognitive Impairment in HIV-Positive Veterans. J Int Assoc Provid AIDS Care. 2015;14(3):197-201.

6. Ustun TB, Kostanjesek N, Chatterji S, Rehm J, World Health O. Measuring health and disability : manual for WHO Disability Assessment Schedule (WHODAS 2.0) / edited by T.B. Üstün, N. Kostanjsek, S. Chatterji, J.Rehm. Geneva: World Health Organization; 2010.

**Supplementary Table 1. Socio-demographic and clinical characteristics concerning physical and mental health of PLWH and age- and sex-matched controls in each recruitment site^1^**

|  | **Hong Kong** | | | **Kuala Lumpur** | | | **Chongqing** | | | **Taipei** | | | **Singapore** | | |
| --- | --- | --- | --- | --- | --- | --- | --- | --- | --- | --- | --- | --- | --- | --- | --- |
| **Variables** | **PLWH** | **Control** | **p value** | **PLWH** | **Control** | **p value** | **PLWH** | **Control** | **p value** | **PLWH** | **Control** | **p value** | **PLWH** | **Control** | **p value** |
| Number | 300 | 99 |  | 207 | 67 |  | 200 | 100 |  | 200 | 100 |  | 97 | 50 |  |
| Demographic characteristics |  |  |  |  |  |  |  |  |  |  |  |  |  |  |  |
| Male | 258 (86.0%) | 84 (84.4%) | 0.776 | 164 (79.2%) | 52 (77.6%) | 0.778 | 134 (67.0%) | 67 (67.0%) | 1.000 | 197 (98.5%) | 99 (99.0%) | 0.722 | 94 (96.9%) | 48 (96.0%) | 0.774 |
| Age (years) | 56.9 ± 11.2 | 57.3 ± 11.7 | 0.721 | 51.8 ± 9.9 | 50.0 ± 11.4 | 0.211 | 56.3 ± 9.2 | 56.8 ± 9.2 | 0.678 | 49.1 ± 8.5 | 49.4 ± 10.6 | 0.831 | 51.2 ± 9.2 | 50.0 ± 9.6 | 0.440 |
| Socio-behavioural factors |  |  |  |  |  |  |  |  |  |  |  |  |  |  |  |
| Married | 102 (34.0%) | 65 (65.7%) | <0.001 | 67 (32.4%) | 53 (79.1%) | <0.001 | 144 (72.0%) | 87 (87.0%) | 0.004 | 15 (7.5%) | 53 (53.0%) | <0.001 | 15 (15.5%) | 37 (74.0%) | <0.001 |
| No employment | 114 (38.0%) | 31 (31.3%) | 0.230 | 57 (27.5%) | 11 (16.4%) | 0.064 | 106 (53.0%) | 57 (57.0%) | 0.512 | 28 (14.0%) | 19 (19.0%) | 0.261 | 20 (20.6%) | 6 (12.0%) | 0.194 |
| Ever smoker | 122 (40.7%) | 26 (26.3%) | 0.010 | 105 (50.7%) | 14 (20.9%) | <0.001 | 95 (47.5%) | 51 (51.0%) | 0.567 | 98 (49.0%) | 43 (43.0%) | 0.326 | 53 (54.6%) | 11 (22.0%) | <0.001 |
| Current smoker | 61 (20.3%) | 11 (11.1%) | 0.039 | 60 (29.0%) | 6 (9.0%) | 0.001 | 41 (20.5%) | 17 (17.0%) | 0.469 | 59 (29.5%) | 22 (22.0%) | 0.168 | 32 (33.0%) | 3 (6.0%) | <0.001 |
| Alcohol use more than once per month | 48 (16.0%) | 21 (21.2%) | 0.234 | 23 (11.1%) | 7 (10.4%) | 0.880 | 38 (19.3%) | 17 (17.0%) | 0.631 | 53 (26.5%) | 28 (28.0%) | 0.783 | 12 (12.4%) | 9 (18.0%) | 0.356 |
| Substance use | 36 (12.0%) | 9 (9.1%) | 0.428 | 45 (21.7%) | 6 (9.0%) | 0.019 | 5 (2.5%) | 0 (0%) | 0.173^α^ | 74 (37.0%) | 4 (4.0%) | <0.001 | 33 (34.0%) | 1 (2.0%) | <0.001 |
| Lubben social network scale | 11.0 ± 6.0 | 14.2 ± 5.5 | <0.001 | 13.3 ± 5.9 | 15.8 ± 5.5 | 0.002 | 14.4 ± 6.3 | 17.8 ± 5.8 | <0.001 | 12.7 ± 5.9 | 15.3 ± 5.8 | <0.001 | 12.5 ± 6.6 | 17.0 ± 5.1 | <0.001 |
| Physical health |  |  |  |  |  |  |  |  |  |  |  |  |  |  |  |
| Charlson Comorbidity Index | 2 (1,3) | 2 (0, 3) | 0.032 | 1 (0, 2) | 1 (0, 2) | 0.088 | 2 (1,3) | 2 (1,3) | 0.767 | 1 (0,1) | 1 (0,1) | 0.610 | 1 (0, 2) | 1 (0, 1) | 0.314 |
| Presence of any comorbidity | 249 (83.0%) | 70 (70.7%) | 0.008 | 134 (64.7%) | 37 (55.2%) | 0.162 | 175 (87.5%) | 89 (89.0%) | 0.706 | 106 (53.0%) | 52 (52.0%) | 0.870 | 61 (62.9%) | 30 (60.0%) | 0.733 |
| Comorbidities |  |  |  |  |  |  |  |  |  |  |  |  |  |  |  |
| Hypertension | 129 (43.0%) | 33 (33.3%) | 0.089 | 98 (47.3%) | 33 (49.3%) | 0.786 | 14 (7.0%) | 12 (12.0%) | 0.147 | 32 (16.0%) | 8 (8.0%) | 0.055 | 27 (27.8%) | 12 (24.0%) | 0.618 |
| Diabetes | 116 (38.7%) | 17 (17.2%) | <0.001 | 42 (20.3%) | 11 (16.4%) | 0.486 | 16 (8.0%) | 28 (28.0%) | <0.001 | 13 (6.5%) | 2 (2.0%) | 0.092 | 12 (12.4%) | 8 (16.0%) | 0.543 |
| Liver disease | 31 (10.3%) | 1 (1.0%) | 0.003 | 14 (6.8%) | 1 (1.5%) | 0.127^α^ | 5 (27.5%) | 18 (18.0%) | 0.071 | 0 (0%) | 1 (1.0%) | 0.333^α^ | 5 (5.2%) | 0 (0%) | 0.166^α^ |
| Cardiovascular disease | 15 (5.0%) | 6 (6.1%) | 0.682 | 21 (10.1%) | 4 (6.0%) | 0.302 | 54 (27.0%) | 7 (7.0%) | <0.001 | 2 (1.0%) | 2 (2.0%) | 0.603^α^ | 11 (11.3%) | 0 (0%) | 0.016^α^ |
| Osteoporosis | 14 (4.7%) | 2 (2.0%) | 0.377^α^ | 18 (8.7%) | 0 (0%) | 0.009^α^ | 18 (9.0%) | 0 (0%) | 0.002 | 0 (0%) | 0 (0%) | N.A. | 6 (6.2%) | 0 (0%) | 0.096^α^ |
| Chronic pulmonary diseases | 8 (2.7%) | 1 (1.0%) | 0.462^α^ | 5 (2.4%) | 0 (0%) | 0.339 | 11 (5.5%) | 9 (9.0%) | 0.252 | 1 (0.5%) | 0 (0%) | 1.000^α^ | 1 (1.0%) | 2 (4.0%) | 0.267^α^ |
| Chronic kidney disease | 7 (2.3%) | 1 (1.0%) | 0.685^α^ | 11 (5.3%) | 0 (0%) | 0.071 | 4 (2.0%) | 2 (2.0%) | 1.000 | 0 (0%) | 0 (0%) | N.A. | 2 (2.1%) | 1 (2.0%) | 1.000^α^ |
| Cancer | 12 (4.0%) | 4 (4.0%) | 1.000^α^ | 2 (1.0%) | 0 (0%) | 1.000^α^ | 7 (3.5%) | 0 (0%) | 0.100^α^ | 2 (1.0%) | 2 (2.0%) | 0.603^α^ | 1 (1.0%) | 1 (2.0%) | 1.000^α^ |
| Body weight (kg) | 70.1 ± 14.1 | 67.9 ± 10.7 | 0.096 | 66.6 ± 14.9 | 72.2 ± 16.0 | 0.009 | 59.3 ± 10.2 | 62.4 ± 10.6 | 0.014 | 72.7 ± 10.9 | 73.4 ± 12.7 | 0.651 | 70.4 ± 15.3 | 74.3 ± 15.0 | 0.143 |
| Body mass index (kg/m^2^) | 25.1 ± 4.3 | 24.6 ± 3.5 | 0.221 | 23.9 ± 4.9 | 26.0 ± 5.1 | 0.003 | 22.5 ± 3.3 | 23.4 ± 3.1 | 0.022 | 24.8 ± 3.4 | 24.8 ± 3.6 | 0.989 | 24.4 ± 5.1 | 25.6 ± 4.6 | 0.136 |
| Systolic blood pressure (mmHg) | 135.5 ± 20.0 | 133.9 ± 17.2 | 0.453 | 131.8 ± 17.5 | 132.4 ± 17.0 | 0.808 | 126.3 ± 15.8 | 128.8 ± 15.7 | 0.191 | 126.7 ± 13.6 | 126.9 ± 13.7 | 0.903 | 133.0 ± 19.3 | 128.6 ± 16.4 | 0.173 |
| Diastolic blood pressure (mmHg) | 85.0 ± 11.7 | 83.2 ± 9.2 | 0.113 | 83.5 ± 11.8 | 83.4 ± 10.7 | 0.969 | 83.0 ± 11.3 | 83.6 ± 10.5 | 0.685 | 79.9 ± 9.1 | 81.4 ± 9.6 | 0.168 | 75.6 ± 10.3 | 78.1 ± 10.8 | 0.175 |
| Mental health |  |  |  |  |  |  |  |  |  |  |  |  |  |  |  |
| PHQ-9 total score | 3 (0, 7) | 2 (0, 5) | 0.143 | 2 (0, 6) | 2 (1, 4) | 0.332 | 2 (0, 6) | 0 (0, 2) | <0.001 | 4 (1, 7) | 3 (0, 5) | 0.032 | 1 (0, 4) | 1 (0, 3) | 0.579 |
| Moderate or severe depression (PHQ-9 ≥10) | 26 (8.7%) | 2 (2.0%) | 0.025 | 14 (6.8%) | 1 (1.5%) | 0.127^α^ | 21 (10.5%) | 4 (4.0%) | 0.055 | 18 (9.0%) | 5 (5.0%) | 0.220 | 3 (3.1%) | 1 (2.0%) | 1.000^α^ |
| DASS anxiety subscale | 1 (0, 3) | 1 (0, 2) | 0.010 | 2 (1, 4) | 2 (1, 4) | 0.295 | 1 (1, 3) | 1 (0, 1) | <0.001 | 1 (0, 3) | 0 (0, 1) | 0.010 | 1 (0, 2) | 0.5 (0, 1) | 0.375 |
| Anxiety (DASS anxiety subscale ≥8) | 19 (6.3%) | 2 (2.0%) | 0.096 | 15 (7.2%) | 4 (6.0%) | 1.000^α^ | 14 (7.0%) | 1 (1.0%) | 0.025 | 15 (7.5%) | 3 (3.0%) | 0.122 | 3 (3.1%) | 1 (2.0%) | 1.000^α^ |
| DASS stress subscale | 2 (0, 4) | 1 (0, 3) | 0.013 | 3 (1, 6) | 4 (2, 6) | 0.567 | 1 (0, 3) | 0 (0, 2) | 0.004 | 2 (0, 5) | 1 (0, 3) | 0.037 | 1 (0, 4) | 1 (0, 3) | 0.307 |
| Stress (DASS stress subscale ≥15) | 36 (12.0%) | 4 (4.0%) | 0.022 | 25 (12.1%) | 6 (9.0%) | 0.483 | 15 (7.5%) | 1 (1.0%) | 0.018 | 25 (12.5%) | 8 (8.0%) | 0.240 | 9 (9.3%) | 5 (10.0%) | 1.000^α^ |
| Loneliness scale | 3.5 ± 1.8 | 3.3 ± 1.7 | 0.427 | 3.3 ± 1.9 | 2.6 ± 1.8 | 0.005 | 2.7 ± 1.5 | 2.5 ± 1.3 | 0.228 | 3.1 ± 1.9 | 3.2 ± 1.7 | 0.490 | 2.2 ± 1.5 | 1.8 ± 1.4 | 0.126 |
| Cognition |  |  |  |  |  |  |  |  |  |  |  |  |  |  |  |
| International HIV dementia scale | 9.17 ± 2.01 | 10.10 ± 1.63 | <0.001 | 9.61 ± 1.83 | 10.12 ± 1.47 | 0.038 | 8.37 ± 1.95 | 8.78 ± 1.76 | 0.075 | 10.72 ± 1.02 | 10.77 ± 0.98 | 0.700 | 9.95 ± 1.38 | 10.31 ± 1.53 | 0.155 |
| Montreal Cognitive Assessment test | 26 (23, 28) | 27 (25, 29) | 0.001 | 25 (22, 27) | 26 (24, 28) | 0.004 | 20 (16, 24) | 20 (16, 24) | 0.361 | 29 (28, 30) | 29 (28, 30) | 0.592 | 27 (26, 28) | 28 (26, 29) | 0.118 |
| HIV-related variables |  |  |  |  |  |  |  |  |  |  |  |  |  |  |  |
| Duration of HIV (years) | 10.7 (5.5, 17.0) | |  | 13.0 (10.0, 17.7) | |  | 1.0 (0.1, 3.8) |  |  | 13.8 (9.6, 19.1) | |  | 10.3 (6.1, 14.5) | |  |
| Past history of AIDS-defining illness | 104 (34.7%) |  |  | 146 (70.5%) |  |  | 136 (68.0%) |  |  | 83 (41.5%) |  |  | 39 (40.2%) |  |  |
| Current CD4 count (cells/mm^3^) | 558 (372, 748) | |  | 616 (438, 800) | |  | 234 (110, 401) | |  | 617 (500, 788) | |  | 542 (368, 665) | |  |
| Current CD8 count (cells/mm^3^) | 737 (521, 1007) | |  | 736 (527, 940) | |  | 457 (300, 755) | |  | 733 (548, 972) | |  | 752 (559, 999) | |  |
| Current CD4:CD8 ratio | 0.75 (0.49, 1.10) | |  | 0.85 (0.62, 1.20) | |  | 0.42 (0.22, 0.70) | |  | 0.85 (0.63, 1.16) | |  | 0.67 (0.51, 0.95) | |  |
| Current viral load (copies per mL) | 20 (20, 20) |  |  | 34 (34, 34) |  |  | 88 (40, 65300) | |  | 20 (20, 20) |  |  | 20 (20, 20) | |  |
| HIV RNA <50 copies per mL | 69 (89.7%) |  |  | 192 (92.8%) |  |  | 94 (47.0%) |  |  | 190 (95.0%) |  |  | 97 (100.0%) |  |  |
| Receiving anti-retroviral therapy (ART) | 300 (100.0%) | |  | 207 (100.0%) | |  | 164 (82.0%) |  |  | 200 (100.0%) | |  | 97 (100.0%) |  |  |
| Current ART regimen |  |  |  |  |  |  |  |  |  |  |  |  |  |  |  |
| Nucleoside reverse transcriptase inhibitor | 300 (100.0%) | |  | 205 (99.0%) |  |  | 155 (77.5%) |  |  | 198 (99.0%) |  |  | 91 (93.8%) |  |  |
| Non-nucleoside reverse transcriptase inhibitor | 51 (17.0%) | |  | 165 (79.7%) | |  | 84 (42.0%) |  |  | 7 (3.5%) |  |  | 56 (57.7%) |  |  |
| Protease inhibitor | 45 (15.0%) |  |  | 24 (11.6%) |  |  | 12 (6.0%) |  |  | 2 (1.0%) |  |  | 7 (7.2%) |  |  |
| Integrase strand transfer inhibitor | 216 (72.0%) |  |  | 21 (10.1%) |  |  | 66 (33.0%) |  |  | 193 (96.5%) |  |  | 40 (41.2%) |  |  |

^1^ Data are presented as number (proportion), mean ± standard deviation, or median (lower quartile, upper quartile). ^α^ Fisher exact test

**Supplementary Table 2. Indirect effect of HIV on disability mediated by other parameters**

| Parameter | Effect of HIV on the parameter^1^ | Effect of the parameter on disability^1^ | Indirect effect of HIV on disability mediated by parameter^1^ | Proportion of indirect effect contributed by each parameter |
| --- | --- | --- | --- | --- |
| Lubben social support network | -2.7786 | -0.1014 | 0.2816 | 11.39% |
| Charlson comorbidity index | 0.1872 | 0.3010 | 0.0563 | 2.28% |
| PHQ-9 | 1.1228 | 0.8746 | 0.9820 | 39.71% |
| DASS anxiety subscale | 0.7079 | 0.5100 | 0.3610 | 14.60% |
| DASS stress subscale | 0.6473 | 0.3023 | 0.1957 | 7.91% |
| De Jong Gierveld Loneliness Scale | 0.0635 | 0.6319 | 0.0401 | 1.62% |
| International HIV dementia scale | -0.4691 | -0.6433 | 0.3018 | 12.20% |
| Montreal Cognitive Assessment test | -1.1835 | -0.2787 | 0.3298 | 13.34% |

^1^Unstandardized coefficients are shown. All correlations were adjusted for age, sex, site, marital status, smoking, substance use and BMI.
